# Future-oriented careers: what social and emotional capabilities do medical students need? A qualitative analysis

**DOI:** 10.3389/fmed.2026.1732959

**Published:** 2026-06-30

**Authors:** Fan Yang, Tao Yang, Di Wang, Yong Li

**Affiliations:** 1 School of Teacher Education, East China Normal University, Shanghai, China; 2 School of Management, Chengdu University of Traditional Chinese Medicine, Chengdu, Sichuan, China; 3 School of Basic Medicine, Chengdu University of Traditional Chinese Medicine, Chengdu, Sichuan, China; 4 School of Pharmacy, Chengdu University of Traditional Chinese Medicine, Chengdu, Sichuan, China; 5 Center for Higher Education Research and Quality Evaluation, Chengdu University of Traditional Chinese Medicine, Chengdu, Sichuan, China

**Keywords:** competency cultivation, medical education, medical students, qualitative analysis, social and emotional capabilities

## Abstract

**Background:**

Against the backdrop of the continuous development of the medical industry and increasing demands for comprehensive quality in medical professionals, this study aims to identify the social and emotional competencies (SEC) that medical students need for their future career development.

**Methods:**

We conducted semi-structured one-to-one interviews with clinicians (*n* = 7), medical students (*n* = 15), and medical teachers (*n* = 10) from diverse specialties, institutions, and training levels. Interview data were transcribed verbatim and analyzed using NVivo software through a three-level coding process (open, axial, and selective coding) to identify core themes.

**Results:**

Three major themes were identified: (1) Social and emotional competencies required for future careers, including key competencies and their impact on development; (2) The current status and challenges in SEC cultivation, covering manifestations and influencing factors; and (3) Recommendations for training, emphasizing assessment, training subjects, and strategies.

**Conclusion:**

The study clarifies specific SEC requirements for medical careers, reveals complex influencing factors, and underscores the crucial role of these competencies. It is recommended that medical educators and policymakers prioritize systematic SEC training to support medical students’ professional development.

## 1 Introduction

In today’s medical industry, the requirements for medical talent are no longer merely focused on pure professional skills (1). With the rapid development of medical technology and the deepening of medical education, the labor market’s expectations of medical professionals are gradually shifting towards the improvement of comprehensive qualities (2). There is a growing realization that a complete and comprehensive medical education cannot afford to neglect soft skills such as communication, empathy, ethics, and emotional intelligence (3). In recent years, there has been increasing interest in the importance of emotional intelligence for effective clinical practice. Empathy and compassion have always been ideal virtues for doctors (4). Emotional intelligence is not only important for providing good clinical care but also for managing all interpersonal relationships that occur as part of the medical process. Emotional intelligence is crucial for doctors to work effectively as a team among nurses, hospital administrators, and other healthcare professionals (5, 6). Effective communication with the relatives, friends, and families of patients receiving treatment is also important (7). In cases of violence against doctors and healthcare professionals, the vulnerability of patients and their relatives, treatment-related uncertainties, overcrowded hospitals, and overworked healthcare staff are the causes of many such violent incidents (8). They can also be traced back to the lack of emotional intelligence among doctors and healthcare professionals, which exacerbates the situation and drives frustrated patients and their relatives towards violence.

In clinical practice, patients’ expectations of medical services are no longer limited to the treatment of diseases but also include emotional support. Empathetic medical students can better establish trust relationships with patients, gain an in-depth understanding of patients’ pain and needs, and thus significantly improve patients’ treatment compliance and satisfaction (9). In terms of teamwork, the complexity of modern medical services determines that it requires the close cooperation of multidisciplinary teams. As a member of the future medical team, medical students must have a good team spirit, respect the views of people with different professional backgrounds, and communicate efficiently (10). Effective teamwork can not only improve medical efficiency and reduce medical errors but also provide patients with more comprehensive and high-quality medical services (11). In addition, the medical profession has great work pressure. Medical students need to have strong psychological resilience and stress-coping abilities to maintain a positive work attitude and professional enthusiasm and avoid the occurrence of burnout (12).

Social and emotional capabilities are comprehensive concepts, involving various abilities of individuals in social interaction and emotional processing. In 1990, Peter Salovey from Yale University in the United States and John D. Mayer from the University of New Hampshire first proposed the concept of emotional intelligence. They divided emotional intelligence into five aspects: understanding one’s own emotions, managing emotions, self-motivation, recognizing others’ emotions, and handling interpersonal relationships (13). In 1995, Daniel Goleman, a psychology professor at Harvard University in the United States and an expert in emotional intelligence research, published the book *Emotional Intelligence: Why It Can Matter More Than IQ*, which systematically elaborated on emotional intelligence (14). Inspired by Goleman’s theory of emotional intelligence, the Collaborative for Academic, Social and Emotional Learning (CASEL) in the United States proposed, in 2005, five components including cognition, emotion, and behavior as the main content of social and emotional capabilities, namely self-awareness, self -management, social awareness, social skills, and responsible decision-making (15). In 2015, the OECD released a report titled “Skills for Social Progress: The Power of Social and Emotional Skills”, which presented a comprehensive analysis of the role of social and emotional capabilities by the OECD and proposed strategies for improving these skills at the same time (16).

Social and emotional competencies differ from general “soft skills” in both connotation and framework. Soft skills are a broad, loosely defined set of interpersonal and workplace traits (e.g., communication, teamwork, adaptability) often described in practical or occupational contexts. In contrast, social and emotional competencies refer to evidence-based, integrated capacities to process emotions, understand self and others, manage behavior, and make responsible choices—rooted in psychological and educational theory. While the two overlap in practical application (e.g., communication, collaboration), social and emotional competencies are structured, developmental, and learning-targeted, with clear components and measurement frameworks, whereas soft skills remain a general, descriptive label without a unified theoretical system. Social and emotional capabilities are the cornerstone for medical students to establish healthy doctor-patient relationships in the future. There is little global attention paid to the cultivation of medical students’ social and emotional capabilities. Therefore, it is necessary to include the social and emotional capabilities of medical students as part of medical education to train sensitive and understanding doctors for the future.

## 2 Aims and research questions

The primary aims of this study are to:

Identify, with precision, the specific social and emotional capabilities that medical students require for their future careers.
Develop evidence-based teaching strategies and practical activities that medical education can implement to effectively cultivate these social and emotional capabilities.
To achieve these aims, the following research questions will be addressed:
What are the specific social and emotional capabilities, along with their relative importance, that medical students need to possess for successful careers in the medical field?
How can medical educational institutions design and implement teaching strategies and practical activities to systematically cultivate these identified social and emotional capabilities among medical students?

## 3 Methods

### 3.1 Research design

This study employed qualitative methodology and adopted grounded theory as the overall research framework to deeply explore the current situation and inherent characteristics of medical students’ social and emotional capabilities. In-depth semi-structured interview was used as the specific research method for primary data collection. All interview data were transcribed and thematically coded with the assistance of NVivo software. Qualitative methodology is particularly suitable for exploring unexplored educational phenomena and capturing subjective experiential information from participants (17). Furthermore, semi-structured interviews were selected because this flexible data collection method allows researchers to continuously adjust questioning logic according to participants’ real-time responses, which is highly compatible with in-depth qualitative exploration in medical education research (18).

### 3.2 Research subjects

The subjects of this study include relevant stakeholders such as medical students, medical teachers, and clinical doctors. As the core group in medical education, the experiences and needs of medical students during the learning process are crucial for understanding the cultivation of social and emotional capabilities. Medical teachers play a guiding role in the education of medical students, and their teaching methods and concepts influence the cultivation of relevant capabilities of medical students. The feedback from clinical doctors can reflect the performance and needs of medical students’ social and emotional capabilities in the medical workplace.

To efficiently access a diverse pool of participants, we adopted a convenience sampling method, which is particularly advantageous for qualitative research as it allows for quick and easy recruitment of participants who are readily available and meet the basic research criteria, facilitating in-depth data collection within a reasonable timeframe. Regarding the recruitment process, we disseminated recruitment information through various social media platforms, targeting multiple medical colleges and healthcare institutions. Participants were selected based on their direct involvement in medical education or practice, ensuring their relevance to the research topic.

In terms of sample distribution, we strived to cover medical students of different grades, professional directions, educational backgrounds, and levels of clinical experience to ensure a comprehensive capture of the diversity and commonalities of the medical student group in terms of social and emotional capabilities. For medical teachers, we included those from different disciplinary fields, with different teaching years and at different professional title levels to obtain multi-dimensional educational perspectives. For clinical doctors, we cooperated with multiple medical institutions to ensure their representativeness. All participants enrolled in this study had no direct teacher-student supervisory relationship with the research team members. None of the interviewees were current or former direct students taught, managed or supervised by the authors. This independent recruitment arrangement effectively eliminated potential response bias brought by hierarchical affiliation and interpersonal familiarity, and guaranteed the objectivity, authenticity and sincerity of interview statements and reported viewpoints.

Despite the above diversification strategies, it is essential to acknowledge the inherent limitations of convenience sampling and social-media-based recruitment. Online recruitment inevitably generates potential selection bias: participants who actively browse and respond to social media postings typically possess higher online activity and voluntary research participation willingness, which may exclude medically relevant personnel with low social media exposure. Additionally, although we covered diverse demographic backgrounds, the non-random nature of convenience sampling restricts the broader generalizability of the findings. To minimize such bias, we maximized sample heterogeneity across age, occupation, institutional background, and working experience during recruitment, and maintained neutral and non-induced questioning throughout the interview procedure.

Regarding demographic characteristics, information such as the gender, age, educational attainment, and years of work (for medical teachers and clinical doctors) of the research subjects was collected. This is to fully consider the potential impact of these factors on the research results during data analysis. Specifically, there are 10 male participants and 22 female participants. The age distribution is as follows: 16 people are aged 20–29 years old, 9 people are aged 30–39 years old, 4 people are aged 40–49 years old, and 3 people are aged 50–59 years old. The educational backgrounds of the participants include 11 undergraduates, 16 postgraduates, and 5 doctoral students. In terms of identity, there are 10 teachers, 15 medical students, and 7 practicing doctors (see Table 1).

**Table 1:** Basic information of participants.

Items		Number
Gender	Male	10
	Female	22
Age	20–29 years old	16
	30–39 years old	9
	40–49 years old	4
	50–59 years old	3
Educational background	Bachelor’s degree	11
	Master’s degree	16
	Doctoral degree	5
Identity	Teachers	10
	Medical students	15
	Doctors	7

### 3.3 Data collection

Data were collected through semi-structured in-depth interviews. The interview guides contain 11–14 open-ended questions tailored for practicing doctors, medical teachers, and medical students. The interview guide designed open-ended questions around the actual needs of social and emotional capabilities and the current situation of medical education in this regard. Some sample questions include “Please recall, when you were a medical student, were there any courses, activities, or practical opportunities that significantly helped improve your social and emotional abilities?” for doctors, “Which teaching staff or teaching links do you think should bear more responsibility in cultivating medical students’ social and emotional abilities? Why?” for teachers, and “Personally, what kind of support do you expect to receive in the undergraduate or postgraduate stage to help you further develop your abilities?” for medical students. The interviews were conducted online and had an average duration of 30–60 min. This method helps to obtain rich, detailed, and in-depth information (19). All interviews were conducted after obtaining the informed consent of the participants and were recorded throughout, and then transcribed into text for analysis.

In this study, thematic saturation was strictly assessed throughout the interview process. We continuously compared and coded newly acquired interview data. After completing interviews with 26 participants, no new core themes or unique conceptual insights emerged from the subsequent transcripts. The final sample size of 32 participants fully achieved thematic saturation and ensured data robustness, which complied with the qualitative research saturation principle proposed by Guest et al. (19). The additional six participants further verified the stability and consistency of the extracted themes, thereby justifying the adequacy and rationality of the sample size.

This study strictly follows the Helsinki Declaration to ensure the standardization and scientificity of the research process (20). Before starting the research, we actively submitted a detailed ethical review application to the Human Subjects Protection Committee of East China Normal University, comprehensively expounding the purpose, methods, potential risks, and protection measures for participants’ rights and interests of the research. After strict evaluation and deliberation by the Human Subjects Protection Committee of East China Normal University, we successfully obtained ethical review approval (approval number: HR791-2024). This process reflects our high attention to research ethics and lays a solid foundation for the smooth progress of the research.

Throughout the entire research process, we have always integrated ethical principles into every step. From the recruitment of research subjects to the collection, analysis, and use of data, we have strictly followed the procedures and standards stipulated by the ethics committee. For example, when recruiting participants, we fully guaranteed their right to know, introducing to them in plain language the nature, purpose, process, and possible risks and benefits of the research in detail to ensure that they voluntarily participate in the research on the basis of full understanding (21). At the same time, we strictly protect the privacy of participants, anonymize all collected data, and adopt strict data security measures to prevent data leakage or improper use.

### 3.4 Data analysis

The interview text data were coded and analyzed using NVivo software. Adopting the three-level coding method of grounded theory, which includes open coding, axial coding, and selective coding, we first carefully read the interview texts to grasp the data as a whole. The codes were data-driven; during open coding, initial coding nodes were established according to the emerging concepts within the data, and the texts were coded sentence-by-sentence. During this process, continuous comparison and discussion were carried out to ensure the consistency and accuracy of coding (22). With the help of NVivo’s query and visualization tools, key themes and patterns in the data were further identified and analyzed.

To enhance the rigor of the qualitative analysis, we have adopted a series of measures. First, we maintain an audit trail by meticulously documenting the research path. This includes the original data, transcribed texts, meeting records, and process notes during the development of the coding framework (23). Second, after each interview, the interviewers write brief memorandums or reflective notes. These notes are reviewed when necessary, and further reflection is conducted through team discussions (24). Third, as the coding theme structure evolves, we analyze more examples of transcribed texts. This is done to challenge, expand, and optimize the classification coding structure. We generate and test competing hypotheses during research meeting discussions, form or delete code categories as required, and further explore or subdivide existing codes (25). In addition, to guarantee the credibility and stability of the three-level hierarchical coding procedure, all textual materials were independently coded by two trained researchers in strict accordance with the unified coding framework. After completing separate coding work, we compared and cross-checked all coding results. Any inconsistent coding viewpoints and diverged theme classifications were fully discussed and jointly revised until reaching a consensus, so as to effectively ensure satisfactory inter-rater reliability and reduce subjective coding bias in qualitative data analysis.

## 4 Results

This study used NVivo software to conduct an in-depth analysis of the interview data. Through a rigorous coding process, the following three broad themes were extracted, with each theme encompassing specific sub-themes derived from the data. These findings provide rich and valuable information for a deep understanding of issues related to medical students’ social and emotional capabilities. The main themes represent overarching concepts, while the sub-themes are specific elements that emerged inductively during the coding process.

1. Social and emotional capabilities required for medical students’ future careers: This theme focuses on the essential capabilities needed. The sub-themes under it include “The importance of key capabilities” and “The impact on future development”.
2. The current situation and challenges of cultivating medical students’ social and emotional capabilities: It addresses the existing state and hurdles in cultivation. Sub-themes are “The performance of medical students’ social and emotional capabilities” and “Factors influencing the development of medical students’ social and emotional capabilities”.
3. Suggestions for cultivating social and emotional capabilities: This encompasses aspects related to the cultivation process. Sub-themes are “The necessity of assessment”, “The main agents of cultivation”, and “Cultivation strategies”.

### 4.1 Social and emotional capabilities required for medical students’ future careers

#### 4.1.1 The importance of key capabilities

During the discussion on “the importance of key capabilities”, a total of 192 text segments were coded. Among them, “the importance of task ability” was mentioned 38 times, and “the importance of emotional regulation” was mentioned 36 times. Notably, “the importance of collaboration ability” was frequently mentioned by all groups, with a total of 39 coding instances. While a higher coding frequency indicates more frequent mention, it does not solely determine the highest importance; importance was also gauged by the depth of discussion and context. This high frequency, however, does demonstrate the significance of social and emotional capabilities in teamwork.

Medical students were more inclined to discuss “the importance of empathy” and “the importance of cooperation”, which were coded 12 times and 16 times, respectively. Practicing doctors emphasized “the importance of stress resistance” and “the importance of optimism”, coded 17 times and 7 times, respectively. Teachers were more concerned about “the importance of a sense of responsibility”, which was coded 23 times.

**Figure 1:**
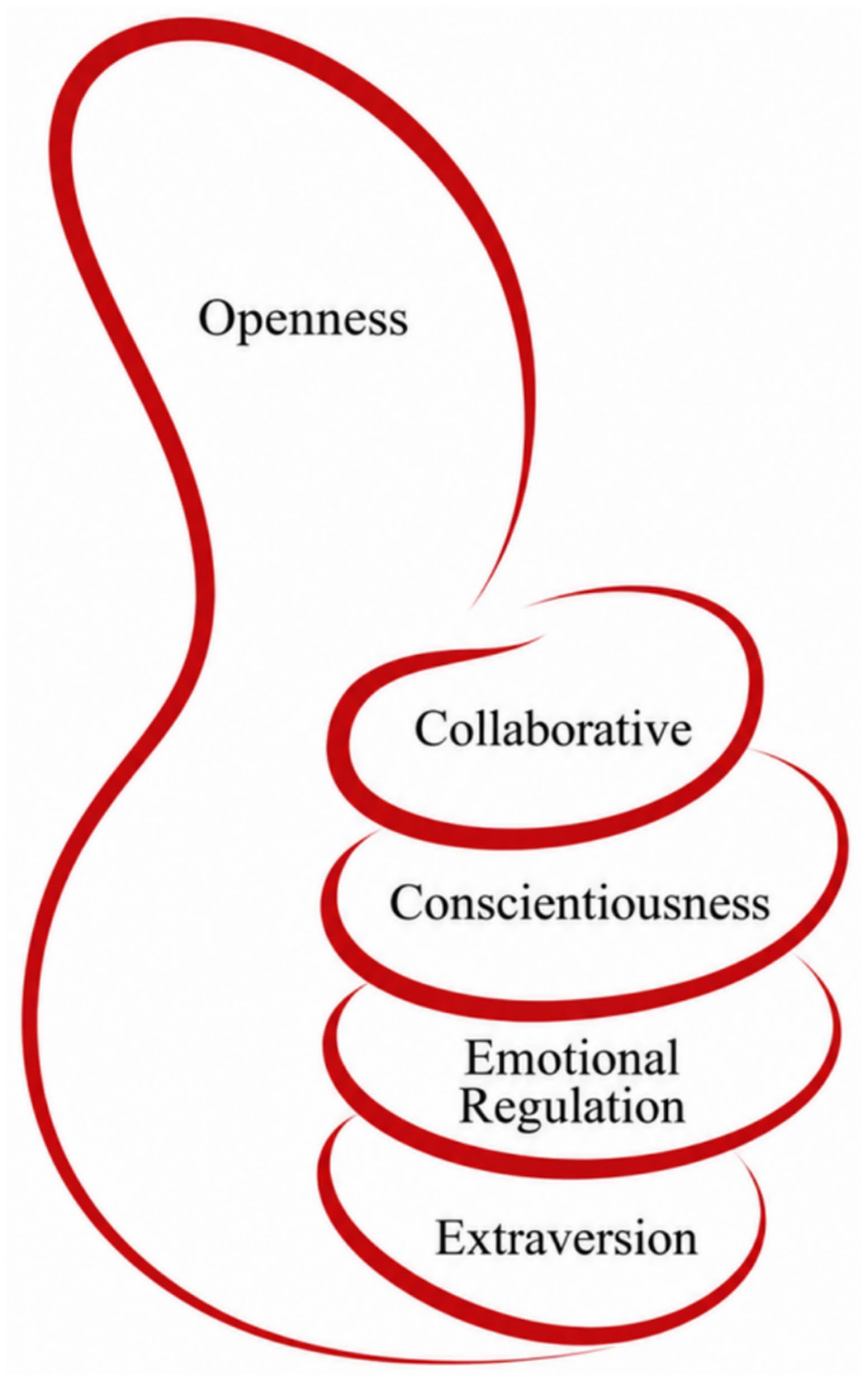
The five-finger model of medical humanistic competence.

By comprehensively analyzing the importance of the 16 sub-capabilities within the five dimensions of social and emotional capabilities for medical students as perceived by the interviewees, a five-finger structure diagram of medical students’ social and emotional capabilities was drawn. The five primary dimensions include openness ability, collaboration ability, task ability, emotional regulation ability, and communication ability (see Figure 1). Each dimension possesses distinct connotations within the medical educational and clinical context. Openness ability reflects individuals’ inclusive cognition, open thinking, and willingness to accept diverse opinions and new knowledge. In medical education and clinical practice scenarios, openness ability covers multiple clear sub-thematic connotations. Specifically, it mainly includes three core sub-dimensions: open-minded thinking, inclusive cognition, and receptive learning attitude. In educational settings, openness is reflected in medical students’ willingness to accept diversified teaching modes, actively absorb interdisciplinary medical knowledge, and openly reflect on their own deficiencies in professional learning and practical training. In clinical working scenarios, this capability manifests as being open to different clinical diagnosis ideas from peers and senior physicians, tolerating different opinions in multidisciplinary diagnosis and treatment discussions, maintaining inclusive attitudes toward patients with different living habits, cultural backgrounds and medical cognition levels, as well as being receptive to clinical feedback, clinical mistakes and targeted professional improvement suggestions. Such open quality helps medical students break professional thinking limitations and better adapt to complex and diversified clinical service environments. Collaboration ability emphasizes interpersonal coordination and mutual assistance within multidisciplinary medical teams. Task ability refers to the individual’s capacity for rational planning, time management, and pressure coping in complex medical work. Emotional regulation ability highlights the stable management of personal emotions and effective emotional interaction with others. Communication ability focuses on accurate expression, effective listening, and empathy in doctor-patient and colleague interaction.

“Cooperation is crucial for a medical team. When a doctor is performing a surgery, it’s definitely not a one-person job. It requires the collaboration of our entire team in the operating room to successfully complete a surgical procedure (S11).”

“In terms of emotional regulation, stress resistance is what I consider to be quite important. Once we start working in a hospital, especially after becoming frontline doctors in clinical practice, we will face a great deal of pressure. Most significantly, the doctor-patient relationship is currently rather tense. There is also pressure from aspects such as interpersonal communication, including interactions with colleagues and superiors. Moreover, for doctors nowadays, clinical performance is not the sole evaluation criterion. There are various other requirements, such as teaching responsibilities, scientific research, and so on. Therefore, the ability to resist stress and regulate anxiety and responses to pressure is extremely important (D06).”

“A sense of responsibility is extremely important for someone who is engaged in a job involving interaction with people. I believe this is the most fundamental ability (T03).”

The interviewees generally believe that for medical students, “task ability,” “emotional regulation,” and “collaboration ability” are more crucial. This echoes the viewpoints in existing literature that these abilities are essential for the career development of medical students (26). In the medical field, task ability involves how individuals manage complex tasks and priorities, which is particularly important for medical students as they need to work in fast-paced and high-pressure environments (27). Emotional regulation ability concerns how medical students handle their emotional reactions and establish effective communication with patients and colleagues (28). The importance of collaboration ability is reflected in the medical team, as medical work usually requires cross-disciplinary cooperation to provide optimal patient care (29). In particular, our research findings emphasize the “importance of collaboration ability,” which is consistent with the emphasis on the role of teamwork in the medical industry in recent studies. For example, a study on medical team communication pointed out that good teamwork can significantly reduce medical errors and improve patient safety (30). Moreover, collaboration ability is also considered a key factor in improving the efficiency and innovation of medical teams (31). In medical education, the cultivation of students’ collaboration ability can be achieved through simulated case discussions, team projects, and cross-professional education (32). In addition, the different focuses of different groups on key abilities also conform to the differences in ability requirements of professional roles mentioned in the literature (33). For example, medical students may be more concerned about how to establish doctor-patient relationships through empathy and cooperation, while practicing doctors may be more concerned about how to maintain emotional stability in high-pressure environments and how to effectively manage the team (34). Teachers may be more concerned about how to cultivate students’ sense of responsibility and self-management through education and assessment (35). These differences highlight the need for personalized and differentiated teaching strategies in medical education to meet the needs of different student groups.

#### 4.1.2 The influence of social and emotional capabilities on the future development of medical students

When discussing “the influence of social and emotional capabilities”, all groups generally agree that social and emotional capabilities have a significant impact on academic achievements and career development. Medical students are more concerned about “the impact on academic achievements”; practicing doctors emphasize “the impact on career development”; and teachers are more concerned about “the impact on professional identity”.

“I think emotional capabilities play a crucial role. There are some doctors who have been practicing for decades, yet they don’t have many regular patients. On the other hand, some doctors have only been working for two or three years, but patients keep coming back to them. This is partly related to our medical skills, but I believe empathy is even more important. When patients feel that they have received real and reliable help and gotten what they need, it makes a big difference. I think this is also related to each person’s sense of responsibility. It is precisely the sense of responsibility and social responsibility that determine every step, every action, and every word we say. They are all closely interconnected (D03).”

“The emotional ability, or ‘*social quotient,*’ demonstrated by medical students in social interactions is one of the hallmarks and fundamental elements of their success. How this ability influences their medical achievements, career development, and professional identity is likely a fundamental, and perhaps even a core, factor (T05).”

“From my personal perspective, I feel that it not only helps me improve to a certain extent in theoretical knowledge, that is, in my academic studies, but also enables me to handle things with a human touch rather than acting like a machine (S12).”

When exploring the impact of social and emotional capabilities (SEC), this study reveals a consensus among all interviewed groups: SEC has a significant impact on the academic achievements and career development of medical students. This finding echoes the viewpoints in existing literature, especially regarding the predictive role of emotional intelligence in the academic performance and future career success of medical students. Research shows that medical students with higher emotional intelligence tend to perform better academically and have smoother career progress (36). In addition, the impact of social and emotional capabilities on professional identity is an area less explored in the existing literature. Our research results provide a new perspective for understanding the comprehensive impact of SEC in medical education. The impact of social and emotional capabilities on professional identity cannot be ignored. Professional identity refers to the degree to which an individual identifies with their professional role, which directly affects their career commitment and job satisfaction (37). In the medical field, a strong professional identity is associated with higher work enthusiasm, better patient care, and a lower turnover rate (38). Our research results emphasize that social and emotional capabilities, especially empathy and self-awareness, are crucial for cultivating medical students’ professional identity. These capabilities enable medical students to better understand and experience patients’ feelings, thus deepening their understanding of the value and goals of the medical profession (39). Moreover, the impact of social and emotional capabilities on the academic achievements of medical students should not be overlooked. Students with higher emotional intelligence are more likely to adopt effective learning strategies, manage learning stress, and form effective study groups with their peers (40). These students also demonstrate higher adaptability and innovation when solving complex problems, which is particularly important for medical education (40). In terms of career development, social and emotional capabilities help medical students build good interpersonal relationships, improve teamwork skills, and play a leadership role in medical teams (41).

### 4.2 The current situation and challenges of cultivating medical students’ social and emotional capabilities

#### 4.2.1 The performance of medical students’ social and emotional capabilities

In the discussion on the “advantages and disadvantages” of the performance of medical students’ social and emotional capabilities, a total of 85 text segments were coded. Among the sub-abilities with relatively prominent performance of medical students’ social and emotional capabilities, medical students are more concerned about “outstanding vitality” and “outstanding cooperation.” Practicing doctors emphasize “outstanding stress resistance” and “outstanding self-control.” Teachers pay more attention to “outstanding sense of responsibility” and “outstanding task ability”. Among the sub-abilities that need further improvement, medical students stress the lack of stress resistance and insufficient emotional regulation ability. Practicing doctors are more concerned about the lack of sense of responsibility and insufficient empathy. The teacher group specifically points out the lack of creativity and insufficient self-control.

“I think that from the kindergarten stage to the higher education stage, there is an increasing emphasis on the cultivation of social and emotional capabilities. In particular, relatively easy-to-develop abilities such as communication and collaboration have been cultivated quite effectively (T06).”

“I feel that medical students these days generally have poor resilience. I’m not referring to their physical resistance, but rather their ability to withstand external pressure. Sometimes, you can neither scold nor criticize them. If you do say something, they might stop working wholeheartedly, or become emotionally unstable and throw tantrums. Such situations are actually quite common (D07).”

“During the clinical internship, standardized training, and postgraduate study stages, it has been found that task ability and emotional regulation ability need to be strengthened. I think this is mainly because the medical profession places extremely high demands on these two aspects (T06).”

Regarding the advantages and disadvantages of medical students’ social and emotional capabilities, our research has revealed the specific perspectives of different groups on these capabilities. These perspectives profoundly reflect the diverse needs and challenges in medical education and practice. Medical students tend to emphasize “vitality” and “cooperation.” This may be related to their emphasis on teamwork and active participation during the learning process. Vitality represents the enthusiasm and motivation of medical students, which is crucial for maintaining long-term learning motivation and coping with the challenges in medical education (42). Cooperation, on the other hand, is an indispensable part of the medical profession. Since medical work is essentially teamwork, it requires coordination and communication among professionals from different fields (29). Practicing doctors and teachers are more concerned about “stress resistance” and “sense of responsibility.” This may be related to their profound understanding of the practical requirements and ethical responsibilities of the medical profession. Stress resistance is a key factor in the success of a medical career. Doctors often face life-and-death decisions and high-pressure situations, and they need to remain calm and rational (43). The sense of responsibility is the core of medical professional ethics. Doctors are responsible for the health and lives of their patients, and this sense of responsibility drives them to adhere to high-standard professional conduct in practice (44). These differences may reflect the different experiences and expectations of various groups in medical education and practice. Medical students may be more focused on how to adapt to the learning environment and future professional requirements through cooperation and vitality, while practicing doctors and teachers are more concerned with how to cultivate students’ stress resistance and sense of responsibility to ensure that they are competent in future medical practice. Consistent with the possible deficiencies in emotional intelligence among medical students mentioned in the literature, our research findings further emphasize the need for special attention to these areas in medical education (39).

#### 4.2.2 Factors influencing the development of medical students’ social and emotional capabilities

In the discussion on the “factors both inside and outside education” that affect the development of medical students’ social and emotional capabilities, a total of 81 text segments were coded. Among them, “educational factors” were mentioned 37 times, while “external factors” were mentioned 22 times. Medical students are more inclined to discuss “academic pressure” and “professional competitions”; practicing doctors emphasize the influence of “social environment”; teachers are more concerned about “lack of educational concept” and “design of training programs”.

“In our school, I think we are quite weak in this regard. Neither the leadership nor many of our teachers have noticed this issue. You know, the influence of a teacher on students’ growth is very important. A teacher’s charisma matters more than their knowledge (T03).”

“It seems that the existing talent cultivation plan in our school definitely emphasizes science over liberal arts. Under the influence of this plan, all our curriculum construction also focuses more on science than liberal arts. This whole set of influence permeates throughout the entire talent cultivation plan, and this kind of thing is lacking in the plan (T04).”

“I think it’s mainly that society has very high expectations of this profession, but the material conditions provided are relatively scarce. I’m a bit worried that if society tries to guide this kind of value orientation, it might mislead the values. After all, the medical profession essentially requires a great deal of dedication (D04).”

The discussion on factors influencing the development of medical students’ social and emotional capabilities reveals the complexity of factors both within and outside the education system. In this study, medical students mostly discussed “academic pressure” and “professional competitions,” which reflect the direct challenges they face in the process of medical education. Academic pressure not only affects the emotional state and mental health of medical students but may also impact the development of their emotional intelligence and empathy (45). Professional competitions may lead to the damage of cooperation among students, thus affecting the cultivation of teamwork skills (46). Practicing doctors and teachers focus on “social environment” and “lack of educational concept”, highlighting the influence of social cultural background and internal factors of the education system on the development of medical students’ social emotional capabilities. The social environment provides the context for medical students to learn and imitate, while the lack of educational concept may lead to insufficient cultivation of medical students’ professional ethics and sense of responsibility (47). The influence of pressure and competition in medical education on emotional development mentioned in existing studies is consistent with our findings. Research shows that continuous pressure and competitive environment may lead to burnout among medical students, affecting their empathy and quality of patient care (42). In addition, our research results expand this discussion by emphasizing the importance of internal factors within the education system, such as educational concept and training program design, for the development of medical students’ social emotional capabilities. The educational concept is the core of medical education, guiding the setting of educational goals and the choice of teaching methods (48). The lack of a clear educational concept may lead to the neglect of the cultivation of emotional intelligence and social skills in educational practice. The design of the training program directly affects the learning experience and ability development of medical students. A comprehensive and balanced training program should include the cultivation of emotional intelligence and social capabilities to promote the all-round development of medical students (49).

### 4.3 Suggestions for cultivating social and emotional capabilities

#### 4.3.1 Emphasize the assessment of medical students’ social and emotional capabilities

In the discussion on “the necessity of ability assessment”, a total of 15 text segments were coded. Among them, “support for assessment” was mentioned 11 times, while “non-support for assessment” was mentioned 4 times. Medical students are more inclined to support “ability assessment”; practicing doctors have different views on “ability assessment”; teachers are more concerned about the implementation details of “ability assessment.”

“It’s good to conduct an assessment. From an educational perspective, the assessment serves as a kind of guidance. It can make students pay attention to these aspects in the context of education (T03).”

“I think it’s necessary. I’m wondering if social and emotional capabilities can be measured in a way similar to, say, the MBTI. Through such a test, one can roughly understand which dimensions are relatively strong and which ones are a bit lacking. Then they’ll know which areas to improve on and how to develop their strengths. It would probably be quite interesting. It’s like gaining an additional understanding of oneself (D06).”

“I don’t think we should turn this into an exam. I believe it should mainly be about self-understanding. If it becomes an exam, it will be very utilitarian. Everyone will know how to answer just to get a good score, and the results won’t be very authentic (D06).”

“Actually, it’s very difficult. It’s extremely challenging to compile an effective question bank (T04).”

Regarding the necessity of conducting assessments of medical students’ SEC, this study has found that medical students generally support SEC assessments, while practicing doctors and teachers are more concerned about the implementation details of these assessments. This finding echoes the discussions in existing literature on the needs and challenges of emotional intelligence assessment in medical education (45). Emotional intelligence, as an important component of social and emotional capabilities, has been widely recognized as having a significant impact on the academic performance and future career success of medical students (36). Therefore, conducting SEC assessments is not only an evaluation of medical students’ capabilities but also an important guarantee for their future career development. The main reason why medical students support SEC assessments is that they recognize the importance of social and emotional capabilities in medical practice. With the increasing requirements for the comprehensive qualities of medical staff in the healthcare industry, medical students are becoming more and more aware of the crucial role of emotional intelligence and social skills in communicating with patients, teamwork, and coping with stress (39). Through assessments, medical students can have a clearer understanding of their own strengths and weaknesses, and thus make targeted improvements. This enhanced self-awareness helps them better adapt to various challenges in their future careers. However, the attention of practicing doctors and teachers to the implementation details of the assessments reflects their profound understanding of the effectiveness and feasibility of these assessments. An effective SEC assessment requires not only scientific assessment tools but also reasonable implementation strategies and a continuous feedback mechanism (50).

#### 4.3.2 The cultivation subjects of medical students’ social and emotional abilities

In the discussion of “staff responsibilities,” a total of 31 text segments were coded. Among them, “counselor responsibilities” were mentioned 9 times, while “full-time teacher responsibilities” and “clinical-teaching teacher responsibilities” were mentioned 8 times each. Medical students pay more attention to the role of “psychological teachers”; practicing doctors emphasize the responsibilities of “administrative staff”; teachers are more concerned about “full-time teacher responsibilities.”

“The first in line should be the student affairs team. Besides that, full-time teachers also have the most interactions with students. The two types of people students have the most contact with are definitely the counselors and the full-time teachers (T01).”

“I think the key group among all the staff is definitely the teachers. The key ones are the teachers! This includes our management staff, our logistics service personnel, and frontline staff. That’s because many qualities of these people have a great impact on the students. I don’t think the relatively weaker group is our logistics service staff. Instead, it’s our management and teaching staff (T03).”

“Class mentors and clinical training doctors act more in the capacity of predecessors and those with experience. Meanwhile, they are also the people we interact with the most during the medical practice process. Their ways of handling people and affairs, as well as their medical practice activities, have a subtle influence on us (S14).”

The cultivation of medical students’ SEC is a complex process involving various types of teaching and administrative staff. The discussions in this study have revealed the crucial responsibilities of counselors, full-time teachers, and clinical training teachers in SEC cultivation, which is consistent with the emphasis on the importance of multi-disciplinary teams in medical education in existing literature. These staff members play different roles in the emotional development and professional growth of medical students, but their collective efforts are essential for cultivating students’ comprehensive abilities. Counselors are usually responsible for students’ mental health and well-being and play a fundamental role in SEC cultivation. By providing psychological counseling and stress management techniques, counselors help medical students cope with challenges in their academic and personal lives (51). In addition, counselors lay the foundation for the development of students’ emotional intelligence by promoting the improvement of students’ self-awareness and emotional regulation abilities. While imparting professional knowledge, full-time teachers also bear the responsibility of cultivating students’ SEC. Through teaching methods such as classroom discussions, case analyses, and group collaborations, they cultivate students’ critical thinking and empathy (52). Full-time teachers have a profound impact on students’ professional attitudes and ethical concepts through their teaching styles and role-modeling. Clinical training teachers, or clinical instructors, play an important role in the professional practice of medical students. Through direct guidance in clinical practice, they cultivate students’ communication skills, teamwork abilities, and professional sense of responsibility (29). The feedback and guidance from clinical training teachers are crucial for medical students to transform theoretical knowledge into practical skills. Our research findings highlight the potential role of psychological teachers in SEC cultivation, an area that has been less explored in existing literature. Through specially designed psychological education courses and workshops, psychological teachers can systematically cultivate students’ emotional intelligence abilities such as emotion recognition, emotion management, and social skills (28). Psychological teachers can also provide scientific evidence on the effectiveness of SEC cultivation for medical education through research and evaluation, thus promoting the improvement and development of educational practices.

#### 4.3.3 Strategies for cultivating medical students’ social and emotional capabilities

In the discussion on the “policy design” for cultivating medical students’ social and emotional capabilities, medical students are more inclined to discuss “school atmosphere” and “all courses.” Practicing doctors emphasize the importance of “top-level design”, while teachers focus more on “reform of assessment methods” and “design of specialized courses”.

“In my understanding, social and emotional capabilities must be cultivated in specific activities and can only be demonstrated in such activities. Without activities, there is no way to showcase these abilities, cultivate them, or make them evident. This gives the cultivation of these abilities its unique characteristics (T05).”

“Creating an atmosphere is indeed crucial. If schools promote these aspects in daily life, it would be even more meaningful (S12).”

“The core of university education, without a doubt, is the classroom. However, in terms of teaching top-level design, we need to figure out how to achieve the cultivation of medical professionals within the classroom. We are extremely short of the emotional intelligence required for medical careers. We lack the ability to translate professional knowledge into language that patients and the public can understand. Even the basic skills of translating professional knowledge into layman’s terms seem to be severely lacking (T05).”

In terms of the reform strategies for the cultivation of medical students’ SEC, this study has revealed the expectations of different groups regarding policy design, providing valuable insights for the formulation of medical education policies. Medical students and teachers tend to focus on school-level reforms, which may be related to their direct experience of the educational environment and teaching practice. They recognize that by improving the school atmosphere, curriculum design, and assessment methods, the cultivation of SEC can be effectively promoted (50). For instance, a supportive and inclusive school atmosphere can encourage students to express their emotions, build trust, and foster teamwork (53). Moreover, innovative curriculum design, such as scenario-based learning and interdisciplinary learning, can provide opportunities for students to practice SEC and enhance their application abilities (54). On the other hand, practicing doctors emphasize the importance of top-level design, which reflects their concern about the overall structure and direction of medical education. Top-level design involves the overall planning and strategic deployment of medical education policies, including setting educational goals, formulating educational standards, and evaluating educational outcomes (48). Practicing doctors may be more aware that the policy design of medical education needs to be aligned with the actual needs and future trends of the medical industry to ensure that graduates can meet industry standards and patient expectations (55).

## 5 Discussion

### 5.1 Significance of the research findings

Our study identified that SEC play a pivotal role in medical students’ professional identity formation. Students also reported significant difficulties in stress management, which underscores the importance of developing SEC in medical education. Additionally, we found divergent views among doctors regarding the assessment of SEC, indicating a need for further exploration in this area.

When comparing these findings with existing literature, several notable aspects emerge. Previous research has also highlighted the importance of SEC in healthcare settings, but our study provides more detailed insights into the specific capabilities that medical students need and the challenges they face in developing them. For example, while some studies have mentioned the impact of communication skills on patient outcomes, our research specifically shows how empathy, a key component of SEC, directly affects patients’ treatment compliance. This adds to the existing body of knowledge by offering a more nuanced understanding of the relationship between SEC and patient care.

The results have prominently demonstrated that SEC plays a central and indispensable role within the medical context. In the realm of medical practice, SEC not only directly impacts the academic achievements and career advancements of medical students but also exerts an indirect influence on patients’ treatment experiences and therapeutic outcomes. Medical students possessing a higher level of SEC are better equipped to comprehend patients’ needs and establish efficacious doctor-patient communication. This conclusion is consistent with previous studies that have shown that empathetic communication can lead to improved patient satisfaction and treatment adherence. For instance, a study by Haskard Zolnierek and DiMatteo (56) found that patients were more likely to follow treatment plans when they felt understood by their healthcare providers.

This result has profound implications for existing medical education. Firstly, medical education should attach great importance to the cultivation of SEC and integrate it into the core objectives of the training program, in line with recommendations from Hsu et al. (57), who emphasize the alignment of SEL domains with ACGME competencies. In terms of curriculum design, specialized curriculum modules for SEC cultivation should be added, such as emotion management, teamwork skills, and empathy training. For example, through scenario simulation courses, medical students can practice their abilities to handle patients’ emotions and teamwork in simulated medical scenarios. At the same time, in the teaching of professional courses, teachers should also focus on guiding students to use SEC to solve practical problems. For instance, in case analysis, the consideration of patients’ psychological factors should be emphasized. In addition, the assessment methods should also be reformed correspondingly. Not only should the mastery of professional knowledge be concerned, but also the performance of SEC should be included in the assessment scope to encourage medical students to actively develop their own SEC, as suggested by Lee et al. (58) and Yoo et al. (59), who developed comprehensive evaluation frameworks for competency-based medical education.

### 5.2 Culturally contextual interpretation of the social and emotional competency dimensions

Distinguished from Western individualism-driven competency frameworks, the five dimensions of medical social and emotional competencies identified in this study carry distinct cultural characteristics under the Chinese collectivist cultural context. In terms of emotional regulation, unlike the Western advocacy of active and open emotional expression, Chinese medical students are culturally encouraged to restrain personal negative emotions to maintain interpersonal harmony and professional etiquette in high-pressure clinical environments. Regarding collaboration ability, Western medical teamwork highlights equal dialogue and decentralized decision-making, while Chinese medical cooperation emphasizes hierarchical respect, collective compliance, and standardized team collaboration under a clear medical rank system. For communication ability, influenced by implicit Eastern communication norms, medical students tend to adopt euphemistic and respectful interaction modes with patients and seniors, rather than the direct expression commonly seen in Western medical communication. In terms of openness, Chinese medical learners exhibit prudent and conservative open cognition; they are willing to accept external suggestions but remain cautious about breaking conventional medical norms, which differs from the radical exploratory openness in Western educational cultures. As for task ability, driven by rigorous academic assessment and public medical responsibility, Chinese medical students prioritize compliance, stability, and task completion accuracy, reflecting a culturally rooted rigorous work attitude. Collectively, these cultural discrepancies shape the unique developmental characteristics of medical students’ social and emotional competencies in China, further verifying the necessity of localized medical competency evaluation.

### 5.3 Research limitations

This study has certain limitations. Firstly, there are limitations in terms of the sample. The research sample mainly focuses on medical students, teachers, and doctors in specific geographical areas or cultural backgrounds. This may result in the research findings not being fully generalizable to other regions or cultural environments. In different cultural backgrounds, there may be differences in the understanding and emphasis on SEC, thus affecting the universality of the research conclusions. Secondly, there may be biases in the data sources. The research mainly relies on the self-reports of the respondents. However, the respondents’ recollections may be biased. They may selectively recall or exaggerate certain experiences and viewpoints, thereby affecting the accuracy of the data. Moreover, as some respondents may be concerned that their answers could have adverse effects on themselves, there may be situations where they do not answer truthfully, which also interferes with the research results to a certain extent.

## 6 Future research directions

Future research can be carried out in multiple directions. On the one hand, there is a need to further develop scales suitable for assessing the SEC of medical students. Current assessment tools may not be able to comprehensively and accurately measure the SEC of medical students. New scales should take into account the characteristics and requirements of medical education, covering all dimensions of SEC, such as emotional intelligence, teamwork ability, etc. To ensure the validity of these new assessment tools, future research could adopt established psychometric methods, including test–retest reliability, construct validity, and content validity. Additionally, AI-based analysis of students’ behaviors in simulated scenarios could provide an objective method for evaluating students’ SEC levels, reducing the subjectivity of traditional assessment. Empirical data from these assessments can drive the reform of SEC cultivation for medical students, enabling the provision of personalized training programs to improve their SEC in a targeted manner.

On the other hand, future research can explore how to integrate SEC cultivation with modern educational technologies, such as virtual reality (VR) and artificial intelligence (AI) technologies. VR can be utilized to create highly realistic medical scenarios, allowing medical students to repeatedly practice and enhance their SEC in a virtual environment. For instance, VR-based role-playing scenarios can simulate complex patient interactions, enabling students to practice empathy and communication skills. AI technology can analyze students’ behavioral performance in these simulated scenarios and provide real-time feedback and improvement suggestions. This integration can offer more immersive and personalized learning experiences, aligning with the goal of targeted SEC cultivation. Nevertheless, the large-scale popularization and application of such technological-assisted training still face prominent practical barriers and resource constraints in current medical education systems. Firstly, high procurement cost, equipment maintenance expenditure and professional technical operation costs raise the financial threshold for most medical colleges. Secondly, there is a general shortage of standardized medical scenario databases and professional teaching designers who are proficient in both medical education and intelligent technology. In addition, limited classroom schedule, fixed curriculum setting and insufficient teacher training also hinder the rapid embedding of VR and AI-assisted SEC training into regular undergraduate and postgraduate medical teaching arrangements. Fully considering these realistic constraints is conducive to putting forward more feasible and phased promotion strategies in subsequent practice.

In addition, interdisciplinary research can be conducted. By integrating knowledge from multiple disciplines such as medicine, psychology, and education, in-depth exploration of the formation mechanism and cultivation strategies of SEC can be achieved, providing a more solid theoretical foundation for medical education reform. These future research directions are closely related to the limitations of our current study, such as sample restrictions and the lack of comprehensive assessment tools, highlighting the need for further exploration in these areas to enhance the understanding and cultivation of medical students’ SEC.

## 7 Conclusion

This study originally aimed to explore the composition dimension, current developmental status, and influencing factors of medical students’ social and emotional competencies, and it attempted to answer the core research questions concerning what core SEC capabilities medical students require in medical contexts and how different stakeholders perceive SEC cultivation. Based on the collected interview data and qualitative analysis, the present research achieved its predefined research objectives. This study offers several significant contributions to the field of medical education. By exploring the SEC of medical students, we have identified five key capabilities (task ability, emotion regulation, collaboration ability, openness ability, and communication ability) crucial for their future careers. In contrast to previous research, our study offers detailed insights into the specific requirements of these capabilities within the medical context, thereby contributing to a more nuanced understanding of how they influence academic success and professional performance.

Regarding SEC cultivation, we have uncovered diverse perspectives among students, teachers, and doctors, along with complex influencing factors ranging from academic pressure to social environment. Notably, we revealed discrepancies in attitudes towards SEC assessment despite a shared recognition of its importance, providing novel insights into the current state of medical education.

The practical implications of our findings are clear. In curriculum design, integrating SEC-focused modules and adopting scenario-simulation and interdisciplinary teaching can enhance students’ practical skills. Reforming assessment methods to include SEC evaluations offers a more comprehensive measure of students’ abilities. Leveraging the roles of various teaching staff creates a holistic cultivation system.

These recommendations stem from our study’s limitations. Our sample’s geographical and cultural constraints, along with potential self-report biases, underscore the need for future research to develop more inclusive assessment tools, explore technology-integrated cultivation methods, and conduct interdisciplinary studies. Together, our findings and proposed improvements lay a foundation for advancing medical education.

## Data Availability

The original contributions presented in the study are included in the article/Supplementary material, further inquiries can be directed to the corresponding authors.

## Supplementary material

The Supplementary material can be found online at: https://www.frontiersin.org/articles/10.3389/fmed.2026.1732959/full#supplementary-material
