# Supplementary material for: Future-oriented careers: what social and emotional capabilities do medical students need? A qualitative analysis
Source: Front Med (Lausanne). 2026 Jun 30;13:1732959. doi: 10.3389/fmed.2026.1732959 (PMC13365043; doi:10.3389/fmed.2026.1732959)
Supplement: Supplementary file 1 [file Table_1.DOCX]

**Interview Outline (Medical School Teachers)**

**I.Current Situation of Medical Students' Social and Emotional Abilities**

1. How do you define the social and emotional abilities that medical students should possess? Please list 1 - 2 of the most important abilities in each dimension and explain the reasons.

2. In your opinion, what are the advantages and areas for improvement in medical students' social and emotional abilities?

3. What specific requirements or expectations does the medical industry have for medical students' social and emotional abilities? Please give examples.

4. How necessary do you think it is to guide medical students to pay attention to social and emotional abilities in medical education? Why?

**II. Influencing Factors**

5. Which internal and external educational factors do you think have promoted or restricted the development of medical students' social and emotional abilities?

6. What are the advantages and challenges in cultivating medical students' social and emotional abilities in medical colleges and universities?

**III. Cultivation Approaches**

7. Please describe which aspects and contents in the current cultivation plan are specifically designed to cultivate medical students' social and emotional abilities?

8. Which courses or teaching activities in the curriculum syllabus are aimed at improving medical students' social and emotional abilities? Please be specific.

9. Which teaching staff or teaching links do you think should bear more responsibility in cultivating medical students' social and emotional abilities? Why?

10. In medical education, how should the balance be struck between professional skills education and the cultivation of social and emotional abilities? Please share your views or experiences.

11. How do you think the policy level should be designed to more effectively promote the cultivation and assessment of medical students' social and emotional abilities?

**IV. Guiding Results**

12. What is the impact of social and emotional abilities on medical students' academic achievements, career development, and professional identity? Please provide specific insights.

13. Do you support the addition of an assessment of medical students' social and emotional abilities in the medical licensing examination or the health professional examination? Please elaborate on your reasons.

**V. Others**

14. Besides the issues discussed above, what other aspects do you think are crucial for the cultivation of medical students' social and emotional abilities?

**Interview Outline (Medical Students)**

**I.Current Situation of Medical Students' Social and Emotional Abilities**

1. Do you understand social and emotional abilities? If you were to choose 1 - 2 of the most important abilities in each dimension for doctors, what would you choose? How should they be defined for medical students?

2. Based on your observation, which abilities are more prominent among your classmates? Which abilities need to be improved?

**II. Influencing Factors**

3. What factors do you think affect the development of medical students' social and emotional abilities?

**III. Cultivation Approaches**

4. Do you consciously try to improve these abilities? What are the general ways?

5. In the current professional cultivation plan, which aspects and contents reflect the cultivation goals and requirements of social and emotional abilities?

6. Which personnel do you think should bear more responsibility for the cultivation of medical students' social and emotional abilities? Why?

7. Which links do you think should be designed more for the cultivation of medical students' social and emotional abilities? Why?

8. Personally, what kind of support do you expect to receive in the undergraduate or postgraduate stage to help you further develop your abilities?

**IV. Guiding Results**

9. What do you think is the impact of social and emotional abilities on the personal development of medical students?

10. Besides the content discussed above, what other important aspects that have not been mentioned do you think are necessary to become an excellent doctor?

**V. Others**

11. Besides the issues discussed above, what other aspects do you think are crucial for the cultivation of medical students' social and emotional abilities?

**Interview Outline (Doctors)**

**I.Current Situation of Social and Emotional Abilities**

1. Do you understand social and emotional abilities? If you were to choose 1 - 2 of the most important abilities in each dimension for medical students (future doctors), what would you choose? How should they be defined for medical students?

2. Based on your observation of the doctor group, what abilities are more prominent among them currently? Which abilities need to be strengthened?

3. What specific requirements or expectations does the medical industry have for doctors'/medical students' social and emotional abilities?

4. Do you think it is necessary to guide doctors/medical students to pay attention to their social and emotional abilities? Why?

**II. Influencing Factors**

5. What factors do you think affect the development of medical students' social and emotional abilities? Are there any restrictive and beneficial conditions in reality?

**III. Cultivation Approaches**

6. Please recall, when you were a medical student, were there any courses, activities, or practical opportunities that significantly helped improve your social and emotional abilities?

7. Which personnel do you think should bear more responsibility for the cultivation of medical students' social and emotional abilities? Why?

8. Which links do you think should be designed more for the cultivation of medical students' social and emotional abilities? Why?

9. How do you think the policy level should be designed to promote the implementation of medical students' social and emotional abilities?

**IV. Guiding Results**

10. How do you view the important role of social and emotional abilities in the (professional) development of medical students/doctors?

11. Do you think it is possible or necessary to add an assessment of medical students'/doctors' social and emotional abilities in the future medical licensing examination or the health professional examination? Why?

12. What other important issues that have not been mentioned above do you think are important for the cultivation of medical students?

**V. Others**

13. Besides the issues discussed above, what other aspects do you think are crucial for the cultivation of medical students' social and emotional abilities?
